# Supplementary material for: Evaluation of the Difference in the Content of Essential and Non-Essential Elements in Wild Boar and Swine Tissues Sampled in the Same Area of Northern Italy
Source: Animals (Basel). 2024 Mar 7;14(6):827. doi: 10.3390/ani14060827 (PMC10967638; doi:10.3390/ani14060827)
Supplement: Supplementary file 1 [file animals-14-00827-s001.zip › animals-2785796-supplementary.pdf]

## Supplementary materials

Table S1: Distribution of ages

| | Mean | Median | Min-Max |
|---|---|---|---|
| <2y.o. | 1.046154 | 1 | 0.6-2 |
| >2y.o. | 3.52381 | 3 | 3-5 |

Table S2: Swine diet composition.

| **Ingredients** | **[%]** |
|---|---|
| Corn | 44.02 |
| Integral barley flour | 14.67 |
| Wheat bran | 12.72 |
| Soya extract flour | 8.8 |
| Carob | 7.83 |
| Wheat meal | 7.83 |
| Brown molasses | 1.96 |
| Calcium carbonate | 1.17 |
| Sodium bicarbonate | 0.49 |
| Sodium chloride | 0.49 |
| L-Lysine | 0.02 |
| TOT | 100 |

Figure S1: Calibration curve for Crhomium.

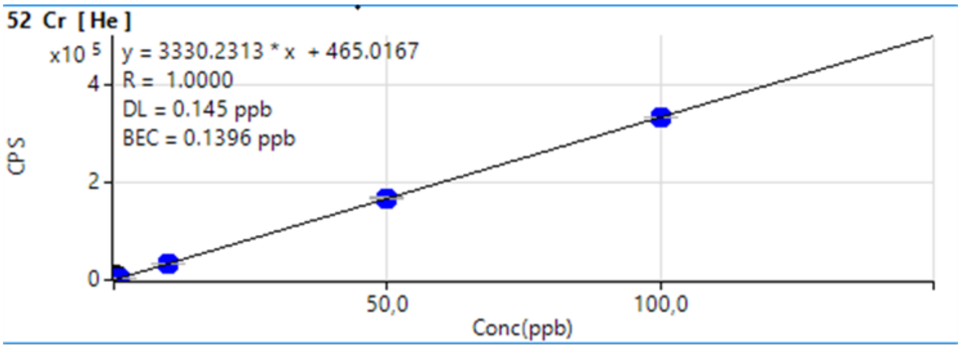


Figure S2: Calibration curve for Arsenic.

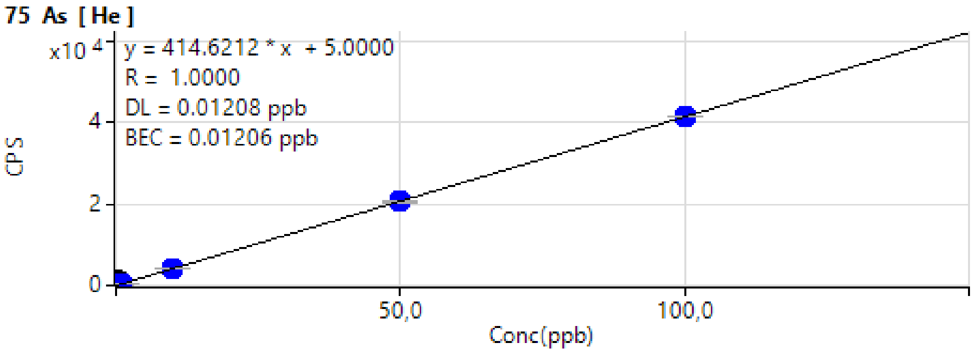

Figure S3: Calibration curve for Selenium.

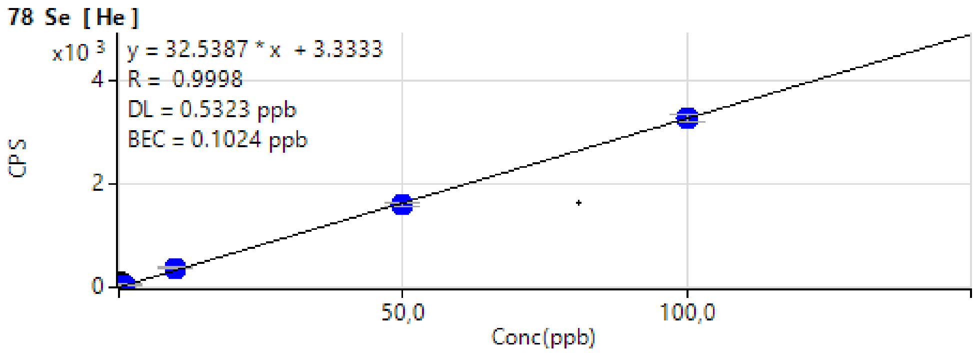


Figure S4: Calibration curve for Cadmium.

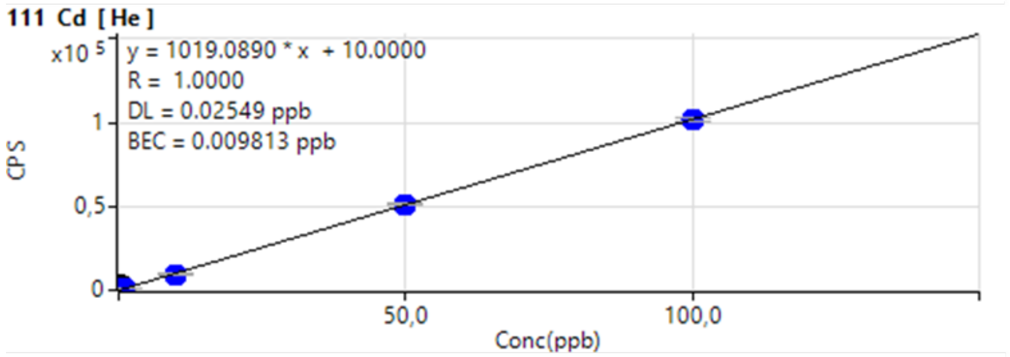


Figure S5: Calibration curve for Lead.

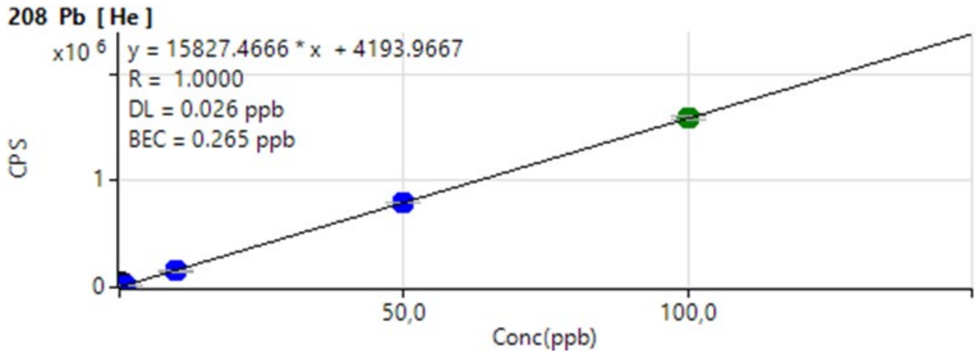

Table S3: Comparison of concentrations (μg·kg$^{-1}$) of trace and potentially toxic elements in liver of wild boar according to the two different geographic areas PV and PC

| Element | Area | Mean ± SD | Min - Max | 25th | Mediana | 75th | p-Value |
|---|---|---|---|---|---|---|---|
| Li | PV | N.D. | N.D. | N.D. | N.D. | N.D. | |
| | PC | N.D. | N.D. | N.D. | N.D. | N.D. | |
| Be | PV | N.D. | N.D. | N.D. | N.D. | N.D. | |
| | PC | N.D. | N.D. | N.D. | N.D. | N.D. | |
| Al | PV | 4016.32 ± 478.02 | 3286.99 - 5059.56 | 3672.81 | 4054.09 | 4242.75 | > 0.05 |
| | PC | 3999.27 ± 558.16 | 3155.22 - 5320.32 | 3551.03 | 3996.69 | 4295.41 | |
| V | PV | 47.15 ± 8.97 | 33.79 - 63.22 | 38.77 | 47.04 | 52.86 | > 0.05 |
| | PC | 45.13 ± 9.28 | 33.56 - 68.90 | 39.31 | 41.56 | 50.01 | |
| Cr | PV | 57.06 ± 27.79 | 36.22 - 122.78 | 39.89 | 43.42 | 67.48 | > 0.05 |
| | PC | 43.19 ± 4.47 | 35.91 - 51.65 | 38.56 | 43.84 | 45.77 | |
| Mn | PV | 2371.98 ± 769.24 | 1409.95 - 3595.02 | 1636.22 | 2441.56 | 2995.36 | > 0.05 |
| | PC | 2377.19 ± 918.99 | 1396.41 - 3917.74 | 1584.62 | 2232.57 | 3182.82 | |
| Fe | PV | 210194 ± 73761.08 | 109920.34 - 356189.40 | 152940.88 | 207533.60 | 251237.68 | > 0.05 |
| | PC | 227208.31 ± 83912.81 | 113142.74 - 341860.26 | 163245.67 | 189854.52 | 319272.58 | |
| Co | PV | 27.44 ± 4.14 | 21.69 - 35.40 | 24.52 | 26.58 | 30.07 | > 0.05 |
| | PC | 26.67 ± 4.77 | 21.43 - 36.63 | 23.47 | 24.25 | 30.12 | |
| Ni | PV | 27.02 ± 8.16 | 15.62 - 41.06 | 20.15 | 26.15 | 33.20 | > 0.05 |
| | PC | 28.21 ± 8.33 | 15.59 - 39.21 | 21.80 | 26.99 | 36.94 | |
| Cu | PV | 3543.53 ± 522.92 | 2682.02 - 4453.64 | 3203.49 | 3516.74 | 3849.41 | > 0.05 |
| | PC | 3304.78 ± 603.44 | 2393.55 - 4606.05 | 2826.75 | 3435.96 | 3635.14 | |
| Zn | PV | 30371.08 ± 2874.59 | 25470.24 - 38400.22 | 28649.67 | 29814.06 | 32111.73 | > 0.05 |
| | PC | 31046.19 ± 3136.80 | 26822.84 - 38138.15 | 28442.01 | 31057.21 | 33525.35 | |
| As | PV | 16.42 ± 10.22 | 7.06 - 34.65 | 8.25 | 13.76 | 23.43 | > 0.05 |
| | PC | 11.40 ± 6.34 | 7.22 - 33.89 | 7.76 | 8.77 | 13.81 | |
| Se | PV | 238.09 ± 53.77 | 148.57 - 309.41 | 193.74 | 249.75 | 281.08 | > 0.05 |
| | PC | 240.03 ± 41.71 | 169.72 - 302.94 | 210.20 | 240.49 | 272.97 | |
| Mo | PV | 758.06 ± 182.24 | 534.25 - 1023.87 | 576.53 | 769.26 | 924.70 | > 0.05 |
| | PC | 691.75 ± 201.90 | 481.11 - 1039.25 | 555.38 | 575.11 | 892.81 | |
| Cd | PV | 156.47 ± 111.03 | 43.17 - 313.41 | 47.61 | 206.31 | 254.41 | > 0.05 |
| | PC | 120.87 ± 108.75 | 38.52 - 303.65 | 50.41 | 52.00 | 240.15 | |
| Sb | PV | N.D. | N.D. | N.D. | N.D. | N.D. | |
| | PC | N.D. | N.D. | N.D. | N.D. | N.D. | |
| Ba | PV | 75.46 ± 19.80 | 49.830 - 101.19 | 54.55 | 78.56 | 95.34 | > 0.05 |
| | PC | 70.55 ± 21.81 | 50.03 - 105.29 | 51.81 | 55.65 | 93.60 | |
| Tl | PV | 4.99 ± 4.30 | 0.690 - 11.40 | 1.31 | 2.29 | 10.29 | > 0.05 |
| | PC | 5.93 ± 3.72 | 0.70 - 11.55 | 2.32 | 5.63 | 9.45 | |
| Pb | PV | 51.81 ± 10.99 | 38.94 - 71.39 | 42.59 | 48.77 | 60.02 | > 0.05 |
| | PC | 55.69 ± 11.07 | 39.69 - 71.96 | 47.23 | 56.75 | 64.29 | |

Table S4: Comparison of concentrations (μg·kg$^{-1}$) of essential and non essential elements in muscle of wild boar according to the two different geographic areas PV and PC.

| Element | Area | Mean ± SD | Min - Max | Percentile | | | p-Value |
|---|---|---|---|---|---|---|---|
| | | | | 25th | Mediana | 75th | |
| Li | PV | N.D. | N.D. | N.D. | N.D. | N.D. | |
| | PC | N.D. | N.D. | N.D. | N.D. | N.D. | |
| Be | PV | N.D. | N.D. | N.D. | N.D. | N.D. | |
| | PC | N.D. | N.D. | N.D. | N.D. | N.D. | |
| Al | PV | 5572.23 ± 2177.29 | 2943.25 - 8990.14 | 3700.64 | 4359.30 | 7588.88 | > 0.05 |
| | PC | 5114.68 ± 2023.84 | 2987.73 - 8773.58 | 3208.22 | 3961.37 | 7262.17 | |
| V | PV | 72.52 ± 9.20 | 54.96 - 88.89 | 66.96 | 72.31 | 79.79 | > 0.05 |
| | PC | 72.86 ± 9.33 | 59.87 - 97.24 | 64.90 | 71.82 | 79.30 | |
| Cr | PV | 157.86 ± 105.18 | 45.25 - 329.30 | 65.40 | 99.35 | 262.61 | > 0.05 |
| | PC | 85.48 ± 25.42 | 46.92 - 128.90 | 63.12 | 91.10 | 99.79 | |
| Mn | PV | 628.04 ± 310.85 | 157.88 - 1126.62 | 316.93 | 624.47 | 911.58 | > 0.05 |
| | PC | 587.81 ± 358.94 | 170.25 - 1112.37 | 296.93 | 340.34 | 971.17 | |
| Fe | PV | 31838.52 ± 8702.71 | 20628.17 - 46870.62 | 23477.91 | 29112.98 | 38065.84 | > 0.05 |
| | PC | 27352.26 ± 6989.32 | 20134.93 - 47678.89 | 21909.98 | 25517.20 | 31307.11 | |
| Co | PV | 8.57 ± 6.06 | 3.40 - 20.20 | 4.23 | 5.53 | 14.29 | < 0.05 |
| | PC | 4.37 ± 0.99 | 3.07 - 5.91 | 3.50 | 4.09 | 5.32 | |
| Ni | PV | 81.64 ± 35.03 | 18.54 - 135.52 | 60.48 | 78.82 | 104.52 | < 0.05 |
| | PC | 60.13 ± 32.88 | 17.98 140.74 | 20.57 | 59.82 | 79.04 | |
| Cu | PV | 1836.40 ± 547.72 | 1155.87 - 2909.51 | 1467.84 | 1574.78 | 2318.64 | > 0.05 |
| | PC | 1489.71 ± 190.4 | 1227.15 - 1808.12 | 1293.36 | 1535.15 | 1621.39 | |
| Zn | PV | 35492.01 ± 17614.1 | 16781.84 - 65909.36 | 19389.53 | 23017.68 | 48924.47 | < 0.05 |
| | PC | 19425.25 ± 2505.25 | 15558.35 - 23473.28 | 17825.67 | 18878.96 | 21271.08 | |
| As | PV | 8.81 ± 1.59 | 6.75 - 11.84 | 7.61 | 8.41 | 9.80 | > 0.05 |
| | PC | 8.71 ± 1.39 | 6.90 - 11.22 | 7.54 | 8.47 | 9.55 | |
| Se | PV | 116.63 ± 67.66 | 34.00 - 228.60 | 71.21 | 89.57 | 178.98 | < 0.05 |
| | PC | 72.70 ± 31.35 | 37.51 - 166.31 | 39.86 | 73.70 | 89.36 | |
| Mo | PV | 9.44 ± 4.29 | 0.00 - 14.18 | 8.73 | 10.59 | 12.05 | > 0.05 |
| | PC | 10.98 ± 2.10 | 7.87 - 13.53 | 8.38 | 11.85 | 12.76 | |
| Cd | PV | 11.86 ± 4.07 | 7.16 - 20.66 | 8.81 | 10.04 | 14.82 | > 0.05 |
| | PC | 13.34 ± 5.01 | 7.55 - 21.59 | 9.08 | 13.26 | 15.97 | |
| Sb | PV | N.D. | N.D. | N.D. | N.D. | N.D. | |
| | PC | N.D. | N.D. | N.D. | N.D. | N.D. | |
| Ba | PV | 156.09 ± 102.76 | 54.86 - 337.08 | 62.56 | 117.89 | 245.18 | > 0.05 |
| | PC | 96.30 ± 45.93 | 53.88 - 228.18 | 64.08 | 68.64 | 131.27 | |
| Tl | PV | 0.084 ± 0.21 | 0.00 - 0.61 | 0.00 | 0.00 | 0.00 | > 0.05 |
| | PC | 0.026 ± 0.12 | 0.00 - 0.55 | 0.00 | 0.00 | 0.00 | |
| Pb | PV | 48.88 ± 7.43 | 35.76 - 63.35 | 43.30 | 49.21 | 51.97 | > 0.05 |
| | PC | 50.24 ± 8.83 | 36.79 - 65.91 | 40.59 | 50.42 | 58.56 | |

Table S5: Comparison of concentrations (μg·kg$^{-1}$) of essential and non essential elements in liver of wild boar according to age classes.

| Element | Age class | Mean ± SD | Min - Max | Percentile | | | p-Value |
|---|---|---|---|---|---|---|---|
| | | | | 25th | Median | 75th | |
| Li | < 2 y.o. | N.D. | N.D. | N.D. | N.D. | N.D. | |
| | > 2 y.o. | N.D. | N.D. | N.D. | N.D. | N.D. | |
| Be | < 2 y.o. | N.D. | N.D. | N.D. | N.D. | N.D. | |
| | > 2 y.o. | N.D. | N.D. | N.D. | N.D. | N.D. | |
| Al | < 2 y.o. | 3924.77 ± 468.25 | 3155.22 - 4881.72 | 3583.92 | 3976.28 | 4237.88 | > 0.05 |
| | > 2 y.o. | 4086 ± 554.14 | 3318.43 - 5320.32 | 3676.18 | 4094.93 | 4354.60 | |
| V | < 2 y.o. | 43.06 ± 6.10 | 34.82 - 54.04 | 37.67 | 41.34 | 48.03 | > 0.05 |
| | > 2 y.o. | 47.86 ± 9.60 | 33.56 - 63.22 | 39.98 | 47.43 | 55.36 | |
| Cr | < 2 y.o. | 42.46 ± 4.22 | 36.22 - 49.49 | 38.34 | 42.31 | 45.42 | > 0.05 |
| | > 2 y.o. | 57.03 ± 25.86 | 35.91 - 114.17 | 40.29 | 44.71 | 68.56 | |
| Mn | < 2 y.o. | 2474.52 ± 869.63 | 1396.41 - 3915.63 | 1590.49 | 2433.28 | 3309.35 | > 0.05 |
| | > 2 y.o. | 2274.66 ± 811.75 | 1408.02 - 3917.74 | 1592.59 | 1853.24 | 2752.64 | |
| Fe | < 2 y.o. | 213839.11 ± 81212.15 | 109920.34 - 356189.40 | 150331.42 | 189923.36 | 275494.82 | > 0.05 |
| | > 2 y.o. | 223563.21 ± 77392 | 113142.74 - 333019.24 | 165204.21 | 211863.29 | 291835.96 | |
| Co | < 2 y.o. | 27.37 ± 4.78 | 21.43 - 36.09 | 23.34 | 25.34 | 30.81 | > 0.05 |
| | > 2 y.o. | 26.74 ± 4.14 | 21.77 - 36.63 | 23.71 | 25.32 | 29.09 | |
| Ni | < 2 y.o. | 26.59 ± 8.20 | 15.62 - 41.06 | 20.06 | 25.01 | 32.16 | > 0.05 |
| | > 2 y.o. | 28.63 ± 8.21 | 15.59 - 39.21 | 23.27 | 30.20 | 35.98 | |
| Cu | < 2 y.o. | 3446.61 ± 562.74 | 2621.69 - 4606.05 | 2979.32 | 3511.46 | 3767.52 | > 0.05 |
| | > 2 y.o. | 3401.70 ± 591.55 | 2393.55 - 4453.64 | 2956.64 | 3485.72 | 3771.05 | |
| Zn | < 2 y.o. | 30071.37 ± 2480.87 | 25470.24 - 34791.74 | 28716.08 | 29585.43 | 32018.93 | > 0.05 |
| | > 2 y.o. | 31977.41 ± 4111.65 | 26822.84 - 40956.24 | 29117.34 | 31264.43 | 33525.35 | |
| As | < 2 y.o. | 10.47 ± 3.30 | 7.06 - 15.48 | 7.60 | 8.64 | 13.76 | > 0.05 |
| | > 2 y.o. | 16.20 ± 10.58 | 7.42 - 34.65 | 8.13 | 12.90 | 23.35 | |
| Se | < 2 y.o. | 248.31 ± 39.01 | 163.23 - 302.94 | 224.53 | 251.88 | 275.34 | > 0.05 |
| | > 2 y.o. | 229.82 ± 54.13 | 148.57 - 309.41 | 180.98 | 230.21 | 277.02 | |
| Mo | < 2 y.o. | 713.32 ± 202.47 | 481.11 - 1024.17 | 547.18 | 607.01 | 924.70 | > 0.05 |
| | > 2 y.o. | 736.48 ± 187.13 | 531.50 - 1039.25 | 568.42 | 659.91 | 896.38 | |
| Cd | < 2 y.o. | 131.54 ± 113.32 | 40.69 - 310.91 | 47.87 | 51.76 | 255.45 | > 0.05 |
| | > 2 y.o. | 145.79 ± 108.97 | 38.52 - 313.41 | 49.66 | 55.00 | 239.11 | |
| Sb | < 2 y.o. | N.D. | N.D. | N.D. | N.D. | N.D. | |
| | > 2 y.o. | N.D. | N.D. | N.D. | N.D. | N.D. | |
| Ba | < 2 y.o. | 73.54 ± 21.18 | 49.83 - 101.19 | 54.55 | 59.59 | 95.56 | > 0.05 |
| | > 2 y.o. | 72.47 ± 20.76 | 50.27 - 105.29 | 52.56 | 77.79 | 88.71 | |
| Tl | < 2 y.o. | 6.07 ± 4.03 | 0.73 - 11.55 | 2.24 | 5.49 | 10.29 | > 0.05 |
| | > 2 y.o. | 4.88 ± 3.98 | 0.69 - 11.19 | 1.32 | 4.96 | 7.56 | |
| Pb | < 2 y.o. | 54.73 ± 10.85 | 39.69 - 71.39 | 45.34 | 54.93 | 62.77 | > 0.05 |
| | > 2 y.o. | 52.76 ± 11.48 | 38.94 - 71.96 | 42.59 | 47.32 | 60.96 | |

Table S6: Comparison of concentrations (μg·kg-1) of essential and non essential elements in muscle of wild boar according to age classes.

| | | | | Percentile | | | |
|---|---|---|---|---|---|---|---|
| Element | age | Mean ± SD | Min - Max | 25th | Mediana | 75th | p-Value |
| Li | < 2 y.o. | N.D. | N.D. | N.D. | N.D. | N.D. | |
| | > 2 y.o. | N.D. | N.D. | N.D. | N.D. | N.D. | |
| Be | < 2 y.o. | N.D. | N.D. | N.D. | N.D. | N.D. | |
| | > 2 y.o. | N.D. | N.D. | N.D. | N.D. | N.D. | |
| Al | < 2 y.o. | 5039.20 ± 2084.94 | 2974.10 - 8235.62 | 3407.16 | 3780.52 | 7492.65 | > 0.05 |
| | > 2 y.o. | 5647.70 ± 2098.83 | 2943.25 - 8990.14 | 3647.54 | 6379.95 | 7345.56 | |
| V | < 2 y.o. | 73.14 ± 9.26 | 57.41 - 88.89 | 65.12 | 72.88 | 80.23 | > 0.05 |
| | > 2 y.o. | 70.99 ± 7.44 | 54.96 - 82.46 | 66.86 | 71.71 | 75.40 | |
| Cr | < 2 y.o. | 150.36 ± 94.92 | 58.63 - 324.55 | 93.07 | 99.38 | 253.46 | < 0.05 |
| | > 2 y.o. | 94.22 ± 65.19 | 45.25 - 329.30 | 61.19 | 65.09 | 111.32 | |
| Mn | < 2 y.o. | 515.75 ± 314.58 | 157.88 - 1060.02 | 275.60 | 340.34 | 854.69 | < 0.05 |
| | > 2 y.o. | 700.08 ± 330.93 | 170.25 - 1126.62 | 338.05 | 725.86 | 993.06 | |
| Fe | < 2 y.o. | 29091.32 ± 9648.75 | 20134.93 - 46870.62 | 21823.47 | 23477.91 | 38065.84 | > 0.05 |
| | > 2 y.o. | 30236.82 ± 6381.18 | 20859.10 - 47678.89 | 25554.82 | 30163.97 | 33523.25 | |
| Co | < 2 y.o. | 7.79 ± 6.29 | 3.07 - 20.20 | 3.43 | 4.24 | 14.29 | > 0.05 |
| | > 2 y.o. | 5.29 ± 2.14 | 3.64 - 13.33 | 4.02 | 5.21 | 5.53 | |
| Ni | < 2 y.o. | 74.96 ± 28.28 | 18.00 - 135.52 | 58.52 | 75.90 | 94.42 | > 0.05 |
| | > 2 y.o. | 66.80 ± 41.43 | 17.98 - 140.74 | 20.57 | 68.48 | 94.97 | |
| Cu | < 2 y.o. | 1805.45 ± 469.56 | 1227.15 - 2805.83 | 1502.34 | 1599.14 | 2279.57 | < 0.05 |
| | > 2 y.o. | 1493.91 ± 280.36 | 1155.87 - 2335.55 | 1284.09 | 1470.63 | 1621.39 | |
| Zn | < 2 y.o. | 30797.65 ± 17911.71 | 15558.35 - 65909.36 | 17825.67 | 20037.37 | 47558.72 | > 0.05 |
| | > 2 y.o. | 30653.98 ± 15552.83 | 18125.32 - 64744.32 | 19589.19 | 21271.08 | 48073.32 | |
| As | < 2 y.o. | 8.65 ± 1.49 | 6.75 - 11.84 | 7.27 | 8.35 | 9.40 | > 0.05 |
| | > 2 y.o. | 8.86 ± 1.49 | 6.90 - 11.28 | 7.61 | 8.59 | 10.50 | |
| Se | < 2 y.o. | 112.65 ± 62.35 | 37.51 - 228.60 | 73.70 | 89.57 | 166.51 | < 0.05 |
| | > 2 y.o. | 69.58 ± 33.27 | 34.00 - 166.31 | 39.74 | 71.06 | 88.95 | |
| Mo | < 2 y.o. | 10.90 ± 3.02 | 0.00 - 14.18 | 10.05 | 11.73 | 12.41 | > 0.05 |
| | > 2 y.o. | 9.44 ± 3.78 | 0.00 - 13.53 | 8.25 | 10.08 | 12.40 | |
| Cd | < 2 y.o. | 14.16 ± 4.73 | 8.21 - 21.59 | 9.94 | 13.52 | 17.68 | < 0.05 |
| | > 2 y.o. | 11.02 ± 3.90 | 7.16 - 20.34 | 7.81 | 9.29 | 13.98 | |
| Sb | < 2 y.o. | N.D. | N.D. | N.D. | N.D. | N.D. | |
| | > 2 y.o. | N.D. | N.D. | N.D. | N.D. | N.D. | |
| Ba | < 2 y.o. | 138.18 ± 106.28 | 58.00 - 337.08 | 62.40 | 70.07 | 242.83 | > 0.05 |
| | > 2 y.o. | 123.41 ± 62.56 | 53.88 - 262.46 | 67.13 | 124.32 | 139.82 | |
| Tl | < 2 y.o. | 0.02 ± 0.12 | 0.00 - 0.59 | 0.00 | 0.00 | 0.00 | > 0.05 |
| | > 2 y.o. | 0.08 ± 0.21 | 0.00 - 0.61 | 0.00 | 0.00 | 0.00 | |
| Pb | < 2 y.o. | 48.06 ± 7.82 | 35.76 - 65.91 | 42.43 | 48.39 | 51.73 | > 0.05 |
| | > 2 y.o. | 51.05 ± 8.26 | 37.20 - 63.35 | 45.05 | 53.17 | 59.11 | |

Table S7: Comparison of concentrations (μg·kg-1) of essential and non essential elements in liver of wild boar according to sexes.

| Element | Sex | Mean ± SD | Min - Max | Percentile | | | p-Value |
|---|---|---|---|---|---|---|---|
| | | | | 25th | Median | 75th | |
| Li | Female | N.D. | N.D. | N.D. | N.D. | N.D. | |
| | Male | N.D. | N.D. | N.D. | N.D. | N.D. | |
| Be | Female | N.D. | N.D. | N.D. | N.D. | N.D. | |
| | Male | N.D. | N.D. | N.D. | N.D. | N.D. | |
| Al | Female | 3984.11 ± 442.58 | 3286.99 - 4881.72 | 3681.280 | 3962.66 | 4253.28 | > 0.05 |
| | Male | 4038.36 ± 608.31 | 3155.22 - 5320.32 | 3518.333 | 4057.99 | 4261.40 | |
| V | Female | 44.99 ± 7.95 | 34.82 - 63.22 | 38.997 | 41.80 | 50.30 | > 0.05 |
| | Male | 47.72 ± 10.45 | 33.56 - 68.90 | 38.755 | 47.89 | 55.17 | |
| Cr | Female | 43.33 ± 4.12 | 36.22 - 51.65 | 40.388 | 44.23 | 45.73 | > 0.05 |
| | Male | 60.05 ± 30.52 | 35.91 - 122.78 | 38.505 | 42.79 | 85.52 | |
| Mn | Female | 2429.99 ± 868.19 | 1396.41 - 3917.74 | 1576.633 | 2368.74 | 3272.90 | > 0.05 |
| | Male | 2298.41 ± 810.92 | 1408.02 - 3915.63 | 1602.210 | 2076.13 | 2938.89 | |
| Fe | Female | 218475.40 ± 84165.05 | 109920.34 - 341860.26 | 139606.015 | 194796.24 | 315235.39 | > 0.05 |
| | Male | 219011.57 ± 72418.04 | 113142.74 - 356189.40 | 178715.037 | 208452.63 | 258740.53 | |
| Co | Female | 26.65 ± 4.66 | 21.43 - 36.63 | 23.408 | 24.95 | 29.79 | > 0.05 |
| | Male | 27.61 ± 4.14 | 22.26 - 36.09 | 24.108 | 27.14 | 30.12 | |
| Ni | Female | 27.40 ± 8.46 | 15.62- 39.21 | 18.783 | 26.25 | 36.50 | > 0.05 |
| | Male | 27.89 ± 7.99 | 15.59 - 41.06 | 22.250 | 27.81 | 34.98 | |
| Cu | Female | 3404.56 ± 612.72 | 2532.40 - 4606.05 | 2837.965 | 3496.14 | 3797.72 | > 0.05 |
| | Male | 3451.09 ± 523.70 | 2393.55 - 4284.18 | 3224.195 | 3493.19 | 3720.63 | |
| Zn | Female | 30168.50 ± 2338.57 | 25470.24 - 34342.33 | 28649.670 | 29885.11 | 31740.53 | > 0.05 |
| | Male | 32100.23 ± 4257.92 | 26822.84 - 40956.24 | 28976.622 | 31688.08 | 34055.64 | |
| As | Female | 10.11 ± 3.19 | 7.06 - 15.40 | 7.550 | 8.43 | 13.85 | < 0.05 |
| | Male | 17.64 ± 10.76 | 7.60 - 34.65 | 8.763 | 13.56 | 31.52 | |
| Se | Female | 244.06 ± 39.21 | 164.90 - 302.90 | 220.653 | 245.12 | 276.38 | > 0.05 |
| | Male | 232.19 ± 57.62 | 148.57 - 309.41 | 180.280 | 245.37 | 276.31 | |
| Mo | Female | 706.77 ± 204.23 | 481.11 - 1024.17 | 542.530 | 577.57 | 932.05 | > 0.05 |
| | Male | 749.83 ± 178.93 | 527.59 - 1039.25 | 584.980 | 714.59 | 885.44 | |
| Cd | Female | 131.82 ± 114.09 | 38.52 - 310.91 | 48.353 | 51.88 | 269.93 | > 0.05 |
| | Male | 148.08 ± 106.78 | 40.69 - 313.41 | 49.902 | 130.16 | 231.77 | |
| Sb | Female | N.D. | N.D. | N.D. | N.D. | N.D. | |
| | Male | N.D. | N.D. | N.D. | N.D. | N.D. | |
| Ba | Female | 70.30 ± 20.96 | 49.83 - 101.19 | 52.367 | 58.34 | 93.72 | > 0.05 |
| | Male | 76.72 ± 20.40 | 50.27 - 105.29 | 54.410 | 81.69 | 95.67 | |
| Tl | Female | 5.86 ± 3.93 | 0.70 - 11.55 | 2.235 | 5.43 | 10.53 | > 0.05 |
| | Male | 4.90 ± 4.15 | 0.69 - 11.05 | 0.750 | 3.90 | 9.43 | |
| Pb | Female | 56.31 ± 10.88 | 40.75 - 71.96 | 47.178 | 56.12 | 64.98 | > 0.05 |
| | Male | 50.22 ± 10.63 | 38.94 - 69.64 | 41.940 | 46.59 | 59.66 | |

Table S8: Comparison of concentrations (µg·kg-1) of essential and non essential elements in muscle of wild boar according to sexes.

| Element | Sex | Mean ± SD | Min - Max | Percentile | | | p-Value |
|---|---|---|---|---|---|---|---|
| | | | | 25th | Mediana | 75th | |
| Li | Male | N.D. | N.D. | N.D. | N.D. | N.D. | |
| | Female | N.D. | N.D. | N.D. | N.D. | N.D. | |
| Be | Male | N.D. | N.D. | N.D. | N.D. | N.D. | |
| | Female | N.D. | N.D. | N.D. | N.D. | N.D. | |
| Al | Male | 4988.70 ± 2040.85 | 2974.10 - 7731.17 | 3252.77 | 3686.66 | 7373.78 | > 0.05 |
| | Female | 5609.52 ± 2128.07 | 2943.25 - 8990.14 | 3719.01 | 5362.61 | 7409.48 | |
| V | Male | 75.22 ± 8.14 | 63.72 - 97.24 | 69.43 | 74.19 | 79.67 | > 0.05 |
| | Female | 70.79 ± 9.57 | 54.96 - 88.89 | 62.87 | 71.37 | 77.17 | |
| Cr | Male | 126.69 ± 85.25 | 45.25 - 344.67 | 72.86 | 99.36 | 126.57 | > 0.05 |
| | Female | 145.25 ± 104.69 | 46.92 - 329.30 | 64.53 | 96.38 | 246.49 | |
| Mn | Male | 571.48 ± 348.29 | 170.25 - 1126.62 | 305.99 | 356.83 | 953.88 | > 0.05 |
| | Female | 635.25 ± 324.52 | 157.88 - 1112.37 | 312.36 | 651.45 | 927.63 | |
| Fe | Male | 29086.44 ± 8235.6 | 20859.10 - 47678.89 | 22652.35 | 26209.71 | 33579.50 | > 0.05 |
| | Female | 30802.16 ± 8759.72 | 20134.93 - 47153.56 | 22431.58 | 29981.99 | 37236.11 | |
| Co | Male | 6.74 ± 5.45 | 3.30 - 20.54 | 3.91 | 4.29 | 5.66 | > 0.05 |
| | Female | 7.85 ± 5.66 | 3.07 - 20.20 | 3.83 | 5.38 | 12.99 | |
| Ni | Male | 63.32 ± 30.73 | 18.54 - 127.89 | 54.49 | 61.72 | 75.77 | > 0.05 |
| | Female | 76.56 ± 37.97 | 17.98 - 140.74 | 57.20 | 78.93 | 98.10 | |
| Cu | Male | 1628.78 ± 402.79 | 1155.87 - 2565.77 | 1429.93 | 1545.97 | 1672.55 | > 0.05 |
| | Female | 1824.76 ± 551.12 | 1237.35 - 2909.51 | 1465.91 | 1599.36 | 2289.34 | |
| Zn | Male | 27405.97 ± 15322.57 | 17298.22 - 64744.32 | 18542.67 | 19800.60 | 23270.32 | > 0.05 |
| | Female | 33215.70 ± 17340.79 | 15558.35 - 65909.36 | 18979.04 | 22250.11 | 49030.97 | |
| As | Male | 8.70 ± 1.49 | 6.90- 11.84 | 7.56 | 8.47 | 9.45 | > 0.05 |
| | Female | 8.79 ± 1.50 | 6.75 - 11.23 | 7.63 | 8.44 | 9.98 | |
| Se | Male | 91.56 ± 54.83 | 34.00 - 228.60 | 64.14 | 84.80 | 92.30 | > 0.05 |
| | Female | 109.36 ± 67.39 | 37.51 - 235.83 | 70.91 | 85.77 | 169.63 | |
| Mo | Male | 10.63 ± 3.26 | 0.00 - 14.08 | 8.95 | 11.62 | 12.66 | > 0.05 |
| | Female | 9.44 ± 4.08 | 0.00 - 14.18 | 8.25 | 10.51 | 12.22 | |
| Cd | Male | 13.19 ± 4.60 | 7.16 - 21.58 | 8.95 | 13.39 | 16.28 | > 0.05 |
| | Female | 12.15 ± 4.59 | 7.55 - 21.59 | 8.99 | 9.90 | 15.71 | |
| Sb | Male | N.D. | N.D. | N.D. | N.D. | N.D. | |
| | Female | N.D. | N.D. | N.D. | N.D. | N.D. | |
| Ba | Male | 119.22 ± 85.24 | 53.88 - 334.27 | 62.01 | 66.45 | 137.19 | > 0.05 |
| | Female | 149.29 ± 97.90 | 54.86 - 358.98 | 68.02 | 122.19 | 231.84 | |
| Tl | Male | 0.03 ± 0.14 | 0.00 - 0.61 | 0.00 | 0.00 | 0.00 | > 0.05 |
| | Female | 0.07 ± 0.19 | 0.00 - 0.59 | 0.00 | 0.00 | 0.00 | |
| Pb | Male | 49.37 ± 7.61 | 39.39 - 63.35 | 42.65 | 47.75 | 55.69 | > 0.05 |
| | Female | 49.69 ± 8.59 | 35.76 - 65.91 | 44.55 | 50.20 | 55.36 | |

Table S9: Comparison between concentrations (μg·kg$^{-1}$) of essential and non essential elements in liver of wild boar and swine.

| Element | Species | Mean ± SD | Min - Max | Percentile | | | p-Value |
|---|---|---|---|---|---|---|---|
| | | | | 25th | Median | 75th | |
| Li | Swine | N.D. | N.D. | N.D. | N.D. | N.D. | |
| | Wild boar | N.D. | N.D. | N.D. | N.D. | N.D. | |
| Be | Swine | N.D. | N.D. | N.D. | N.D. | N.D. | |
| | Wild boar | N.D. | N.D. | N.D. | N.D. | N.D. | |
| Co | Swine | 28.25 ± 8.16 | 13.8 - 44.6 | 23.88 | 27.91 | 34.16 | p>0.05 |
| | Wild boar | 27.05 ± 4.42 | 21.43 - 36.6 | 23.67 | 25.33 | 30.15 | |
| Cu | Swine | 16700.72 ± 8969.23 | 7279.6 - 34576.3 | 9115.43 | 16819.97 | 18882.48 | p<0.05 |
| | Wild boar | 3424.16 ± 569.92 | 2393.55 - 4606.1 | 2906.65 | 3495.93 | 3797.72 | |
| Fe | Swine | 336705.3 ± 95226.41 | 186823.61 - 572838.8 | 282535.7 | 318962.8 | 390318.2 | p<0.05 |
| | Wild boar | 218701.16 ± 78400.76 | 109920.34 - 356189.4 | 160623.3 | 206287.8 | 299503.5 | |
| Mn | Swine | 2634.62 ± 977.11 | 1315.67 - 4737.6 | 1656.91 | 2657.7 | 3181.25 | p>0.05 |
| | Wild boar | 2374.59 ± 835.9 | 1396.41 - 3917.7 | 1582.97 | 2301.61 | 3088.43 | |
| Mo | Swine | 2054.06 ± 307.38 | 1421.13 - 2718.3 | 1927.27 | 2076.3 | 2198.3 | p<0.05 |
| | Wild boar | 724.9 ± 192.66 | 481.11 - 1039.3 | 559.68 | 626.64 | 909.07 | |
| Ni | Swine | 58.99 ± 57.33 | 10.55 - 153.4 | 13.15 | 35.05 | 132.07 | p>0.05 |
| | Wild boar | 27.61 ± 8.16 | 15.59 - 41.1 | 21.35 | 26.25 | 35.23 | |
| Se | Swine | 320.24 ± 25.86 | 273.18 - 371.9 | 297.49 | 318.91 | 341.34 | p<0.05 |
| | Wild boar | 239.06 ± 47.47 | 148.57 - 309.4 | 208.26 | 245.12 | 276.96 | |
| Zn | Swine | 66531.84 ± 17365.84 | 36310.75 - 99415.6 | 51416.77 | 66518.42 | 80377.87 | p<0.05 |
| | Wild boar | 31236.67 ± 3644.44 | 25470.24 - 40956.2 | 28716.08 | 30197.55 | 32883 | |
| Al | Swine | 12029.17 ± 9551.85 | 2176.35 - 28549.4 | 2683.48 | 13290.72 | 18216.73 | p>0.05 |
| | Wild boar | 4055.12 ± 585.28 | 3155.22 - 5814.6 | 3650.7 | 3997.19 | 4275.25 | |
| As | Swine | 6.28 ± 1.73 | 3.3 - 10.2 | 5.08 | 6.31 | 7.13 | p<0.05 |
| | Wild boar | 14.49 ± 9.25 | 7.06 - 34.6 | 7.81 | 10.71 | 14.87 | |
| Ba | Swine | 73.94 ± 42.03 | 32.66 - 162.7 | 38.76 | 75.77 | 84.54 | p>0.05 |
| | Wild boar | 73 ± 20.69 | 49.83 - 105.3 | 53.4 | 68.69 | 94.38 | |
| Cd | Swine | 48.34 ± 11.25 | 33.07 - 75.6 | 42.15 | 45.45 | 51.07 | p<0.05 |
| | Wild boar | 138.67 ± 109.89 | 38.52 - 313.4 | 48.35 | 53.87 | 243.55 | |
| Cr | Swine | 116.37 ± 127.26 | 5.52 - 329.1 | 6.71 | 47.8 | 281.41 | p>0.05 |
| | Wild boar | 53.39 ± 24.2 | 35.91 - 122.8 | 39.83 | 44.23 | 48.61 | |
| Pb | Swine | 41.17 ± 12.6 | 17.19 - 63.4 | 35.39 | 44.03 | 50.52 | p<0.05 |
| | Wild boar | 53.75 ± 11.06 | 38.94 - 72 | 42.79 | 54.09 | 61.06 | |
| Sb | Swine | N.D. | N.D. | N.D. | N.D. | N.D. | |
| | Wild boar | N.D. | N.D. | N.D. | N.D. | N.D. | |
| Tl | Swine | N.D. | N.D. | N.D. | N.D. | N.D. | |
| | Wild boar | 5.46 ± 4 | 0.69 - 11.6 | 1.94 | 5.33 | 9.8 | |
| V | Swine | 68.4 ± 21.47 | 35.93 - 113.5 | 51.26 | 66.94 | 72.46 | p<0.05 |
| | Wild boar | 46.14 ± 9.06 | 33.56 - 68.9 | 38.96 | 44.45 | 51.55 | |

Table S10: Comparison between concentrations (μg·kg$^{-1}$) of essential and non essential elements in muscle of wild boar and swine.

| Element | Species | Mean ± SD | Min - Max | Percentile 25th | Percentile Median | Percentile 75th | p-Value |
|---|---|---|---|---|---|---|---|
| Li | Swine | N.D. | N.D. | N.D. | N.D. | N.D. | |
| | Wild boar | N.D. | N.D. | N.D. | N.D. | N.D. | |
| Be | Swine | N.D. | N.D. | N.D. | N.D. | N.D. | |
| | Wild boar | N.D. | N.D. | N.D. | N.D. | N.D. | |
| Co | Swine | 3.31 ± 0.99 | 2.07 - 5.82 | 2.402 | 3.26 | 3.98 | p<0.05 |
| | Wild boar | 7.37 ± 5.53 | 3.07 - 20.54 | 3.905 | 5.19 | 5.86 | |
| Cu | Swine | 2192.87 ± 267.98 | 1696.51 - 2661.15 | 1958.62 | 2234.89 | 2421.32 | p<0.05 |
| | Wild boar | 1740.77 ± 497.28 | 1155.87 - 2909.51 | 1429.928 | 1572.22 | 1786.96 | |
| Fe | Swine | 30023.03 ± 5011.51 | 20760.52 - 42414 | 26520.222 | 29857.6 | 33008.94 | p>0.05 |
| | Wild boar | 30066.85 ± 8479.75 | 20134.93 - 47678.89 | 22563.592 | 28726.03 | 34355.85 | |
| Mn | Swine | 344.42 ± 72.72 | 188.29 - 445.62 | 283.923 | 353.97 | 402.32 | p<0.05 |
| | Wild boar | 607.92 ± 332.26 | 157.88 - 1126.62 | 305.993 | 562.58 | 931.27 | |
| Mo | Swine | 32.14 ± 5.93 | 18.64 - 42.71 | 29.2 | 32.7 | 35.49 | p<0.05 |
| | Wild boar | 9.95 ± 3.76 | 0 - 14.18 | 8.395 | 11.04 | 12.41 | |
| Ni | Swine | 22.18 ± 23.45 | 8.17 - 71.17 | 9.352 | 10.5 | 12.75 | p<0.05 |
| | Wild boar | 70.88 ± 35.28 | 17.98 - 140.74 | 57.045 | 71.78 | 94.83 | |
| Se | Swine | 103.71 ± 13.68 | 84.83 - 130.56 | 92.343 | 102.44 | 112.35 | p<0.05 |
| | Wild boar | 101.72 ± 62.24 | 34 - 235.83 | 66.12 | 84.8 | 99.84 | |
| Zn | Swine | 46536.27 ± 5740.44 | 36327.3 - 59087.38 | 42291.388 | 47079.32 | 50876.56 | p<0.05 |
| | Wild boar | 30725.82 ± 16568.13 | 15558.35 - 65909.36 | 18542.667 | 21048.99 | 47944.67 | |
| Al | Swine | 3191.31 ± 839.44 | 2126.67 - 4965.56 | 2602.878 | 2842.53 | 3995.05 | p<0.05 |
| | Wild boar | 5343.45 ± 2089.05 | 2943.25 - 8990.14 | 3411.582 | 4160.85 | 7398.73 | |
| As | Swine | 6.41 ± 1.47 | 4.4 - 8.85 | 5.13 | 5.77 | 7.82 | p<0.05 |
| | Wild boar | 8.76 ± 1.48 | 6.75 - 11.84 | 7.558 | 8.44 | 9.74 | |
| Ba | Swine | 49.47 ± 17.20 | 19.92 - 80.39 | 35.442 | 44.83 | 65.02 | p<0.05 |
| | Wild boar | 136.41 ± 92.83 | 53.88 - 358.98 | 63.73 | 108.53 | 148.91 | |
| Cd | Swine | 2.50 ± 1.80 | 0.56 - 6.1 | 0.682 | 1.98 | 3.24 | p<0.05 |
| | Wild boar | 12.60 ± 4.57 | 7.16 - 21.59 | 8.877 | 10.43 | 15.8 | |
| Cr | Swine | 25.02 ± 15.67 | 8.73 - 54.67 | 14.027 | 19.9 | 39.67 | p<0.05 |
| | Wild boar | 137.29 ± 96.17 | 45.25 - 344.67 | 64.935 | 99.02 | 138 | |
| Pb | Swine | 41.06 ± 39.81 | 4.97 - 115.45 | 7.09 | 10.59 | 83.22 | p>0.05 |
| | Wild boar | 49.56 ± 8.09 | 35.76 - 65.91 | 43.127 | 49.6 | 55.72 | |
| Sb | Swine | N.D. | N.D. | N.D. | N.D. | N.D. | |
| | Wild boar | N.D. | N.D. | N.D. | N.D. | N.D. | |
| Tl | Swine | N.D. | N.D. | N.D. | N.D. | N.D. | |
| | Wild boar | 0.05 ± 0.17 | 0 - 0.61 | 0 | 0 | 0 | |
| V | Swine | 59.43 ± 7.17 | 46.39 - 74.19 | 53.547 | 60.58 | 64.4 | p<0.05 |
| | Wild boar | 72.69 ± 9.15 | 54.96 - 97.24 | 65.58 | 72.18 | 79.67 | |
